# Robust and Accurate Discrimination of Self/Non-Self Antigen Presentations by Regulatory T Cell Suppression

**DOI:** 10.1371/journal.pone.0163134

**Published:** 2016-09-26

**Authors:** Chikara Furusawa, Tomoyuki Yamaguchi

**Affiliations:** 1 Quantitative Biology Center, RIKEN, 6-2-3 Furuedai, Suita, Osaka 565-0874, Japan; 2 Department of Physics, University of Tokyo, 7-3-1 Hongo, Bunkyo-ku, Tokyo 113-0033, Japan; 3 Immunology Frontier Research Center (WPI-IFReC), Osaka University, 6-2-3 Furuedai, Suita, Osaka 565-0874, Japan; Tokyo Daigaku, JAPAN

## Abstract

The immune response by T cells usually discriminates self and non-self antigens, even though the negative selection of self-reactive T cells is imperfect and a certain fraction of T cells can respond to self-antigens. In this study, we construct a simple mathematical model of T cell populations to analyze how such self/non-self discrimination is possible. The results demonstrate that the control of the immune response by regulatory T cells enables a robust and accurate discrimination of self and non-self antigens, even when there is a significant overlap between the affinity distribution of T cells to self and non-self antigens. Here, the number of regulatory T cells in the system acts as a global variable controlling the T cell population dynamics. The present study provides a basis for the development of a quantitative theory for self and non-self discrimination in the immune system and a possible strategy for its experimental verification.

## Introduction

The problem of self/non-self discrimination is a key issue in immunology. Interactions among a variety of immune cells enable them to recognize and to attack non-self antigens such as bacteria and viruses, whereas they normally remain tolerant to self antigens such as tissues. Self and non-self antigens are recognized by T cells via antigen presentation. Antigen presenting cells (APCs) capture antigens, break them into small peptides, and present them on MHC molecules [1]. T cells interact with the presented antigenic peptides via T cell receptors (TCRs) on their surface, which have structural diversity generated by gene rearrangement [2]. The affinity between antigen and TCR depends on their structures, and controls whether a T cell is activated (i.e., antigen-specific proliferation of T cells) or not [3]. As the number of potential antigens is huge, the number of possible interactions among antigens and TCRs is likewise enormous. An essential question here is how the immune system recognizes unpredictable non-self antigens to which it responds and self antigens to which it is tolerant.

The classical idea of the self/non-self discrimination is that self-reactive T cells, i.e., T cells having TCRs with high affinity to self antigens, are eliminated in their developmental process(here, the term “affinity” is used to describe the relative responsiveness of a TCR to an antigen rather than biophysical properties). The result is that only T cells tolerant to self tissues are allowed to circulate. This assumption is partially true, as T cells that recognize self antigens undergo clonal deletion in the thymus, which is the so-called negative selection process [4]. However, it has been understood that the negative selection is not always complete, i.e., the negative selection only partially deletes self-reactive T cells. Self-reactive T cells exist in healthy individuals, and they are non-activated even in the presence of their cognate self antigens [5]. This fact indicates that the immune response cannot be captured by the reactivity of a single T cell to self/non-self antigens. Rather, the mechanism of self/non-self discrimination should be described by behavior at the cell population level, including various antigens and T cells, activation and suppression of cellular proliferation, and complex cell-cell interactions.

Regulatory T (Treg) cells play an essential role in suppressing aberrant immune reactions against self antigens [6]. Treg cells constitute approximately 10% of peripheral T cells, and depletion of the Treg-fraction from a normal immune system can induce autoimmune diseases [7, 8]. Genetic defects in Treg development also cause fatal autoimmune diseases [9]. These facts show that a substantial number of self-reactive T cells are retained among conventional T (Tconv) cells even after the negative selection in the thymus and further indicate that Treg cells inhibit the proliferation of these self-reactive Tconv cells. Treg cells have as much variety of TCRs as Tconv and are suggested to be selected from self-reactive T cells in the thymus [9]. According to the tracking of T cells with a particular TCR and deep sequencing of TCR genes of T cell subpopulations, TCR repertoires of Treg and Tconv are partially overlapped but not identical, and TCRs of Treg cells tend to have higher affinity to self-antigens than those of Tconv cells [10]. Also, stimulated Treg cells, which are exposed to their specific antigen, are able to suppress proliferation of Tconv with irrelevant antigens [11]. Therefore, despite the necessity of suppression by Treg to avoid autoimmunity, too much intensification of Treg can disturb a beneficial immune response to non-self antigens. Adequate control of Treg population is important to maintain immune response only for non-self antigens.

Given this background, how does the immune system maintain both tolerance to self tissues and responses to any non-self antigens? The important condition here is that the selection of T cells in the thymus is imperfect. Suppose that there is a self antigen and a non-self antigen presented on MHC, and that we can measure affinities of these antigens to all Tconv cells in a body, i.e., obtain affinity distributions. Due to the negative selection in the thymus, the peak of the affinity distribution might be larger for the non-self antigen than for the self antigen, while incomplete negative selection means these two affinity distributions can have a significant overlap. Intuitively, the overlap makes it difficult to set a single threshold affinity level beyond which the Tconv cells are activated only for any non-self antigens. Here, the problem is how robust discrimination of self and non-self antigen is possible by such imperfect selections, which generate only biases in the affinity distributions. Although several mathematical models based on interactions of Tconv, Treg, and APC have been studied [12–15], the variety of antigens and TCRs were not considered in those studies. Thus, the mechanism of immune response which responds only to non-self antigens remains unclear.

In this study, by using a simple stochastic population model of immune cells, we demonstrate that robust discrimination of self and non-self antigens is possible based on slight differences in the affinity distributions to self and non-self antigens, with the aid of suppression by Treg cells. The results provide a novel mechanism of self/non-self discrimination based on a global control of T cell immune activity by Treg cells.

## Model

**Fig 1:**
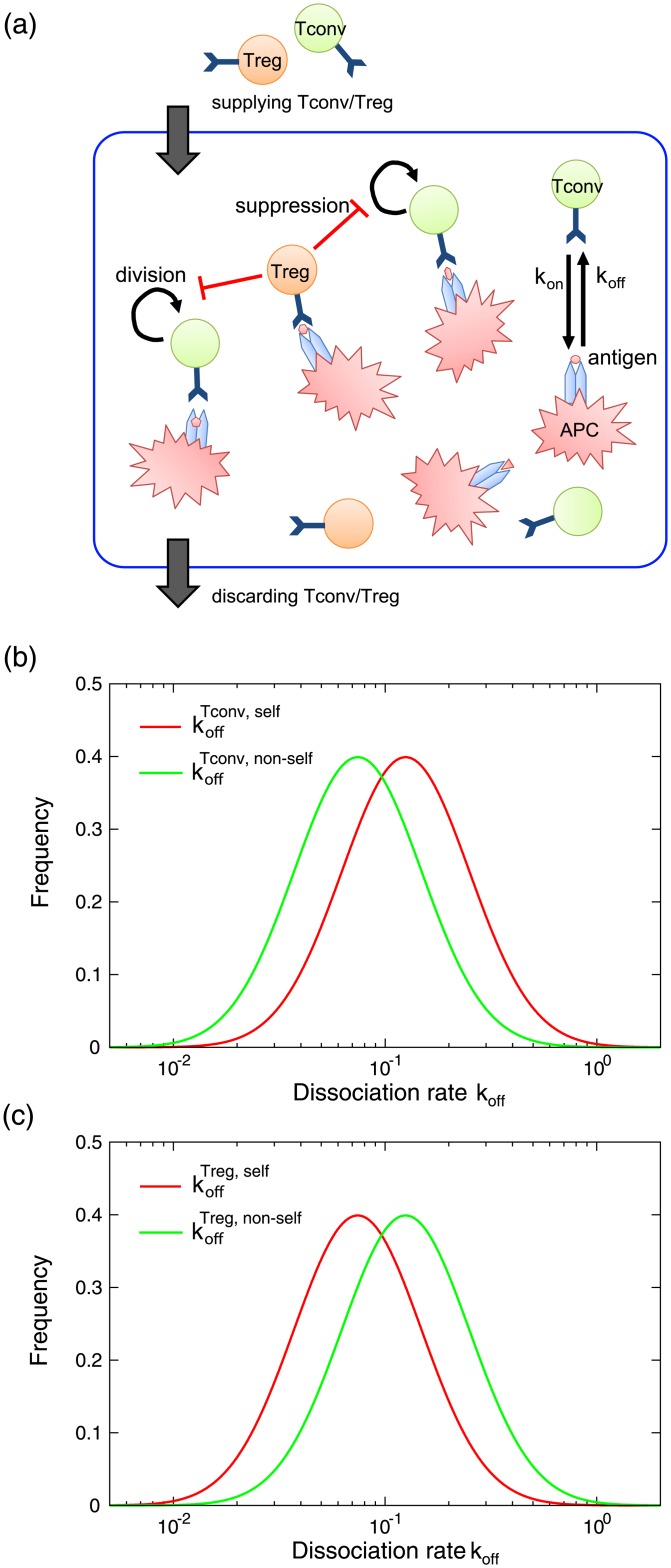
Multicellular model of T cell response. (a) A schematic illustration of the model. Each APC presents a self or non-self antigen. T cells are associated with APC in a stochastic manner, while the dissociation rate *k*_off_ depends on the combination of antigen and TCR expressed on the T cell. These T cells divide only when they are associated with APC, which is suppressed by Treg cells in the environment. Tconv and Treg cells are supplied from outside the system constantly, in a ratio of 9:1, which is based on experimental observation [10]. Simultaneously, T cells which are not attached to APCs are discarded in a constant rate. (b) The distribution of *k*_off_ for Tconv cells. Two distributions of $k_{\text{off}}^{\text{Tconv,self}}$ and $k_{\text{off}}^{\text{Tconv,non-self}}$ of Tconv cells supplied to the system are plotted. The parameters are *μ*^Tconv,self^ = −3, *μ*^Tconv,non−self^ = −3.75 (i.e., Δ^Tconv^ = 0.75), and *σ* = 1. (c) The distribution of *k*_off_ for Treg cells supplied to the system. The parameters are *μ*^Treg,self^ = −3.75, *μ*^Tconv,non−self^ = −3 (i.e., Δ^Treg^ = 0.75), and *σ* = 1.

Fig 1a shows a schematic representation of our model. There are *N* APCs in the environment (e.g., lymph node) which present a self or non-self antigen chosen from *M*^self^ or *M*^non−self^, self or non-self antigen repertoires respectively. Here, we assume that each APC presents a single antigen to simplify the model. This assumption can be relaxed as discussed later. Tconv and Treg cells are continually supplied to the environment from outside the system, and are randomly discarded from the environment at a constant rate. Each T cell expresses a TCR randomly chosen from *K*^Tconv^ repertoire for Tconv cells and *K*^Treg^ repertoire for Treg cells.

In this model, the difference in the affinity between TCRs and presented antigens is represented by the difference in dissociation rate constant (*k*_off_) in analogy with chemical kinetics, which represents the probability that a T cell attached to an APC is dissociated per unit time. For simplification, we assume that each free T cell binds to an antigen on an APC in a stochastic manner with a fixed association rate constant *k*_on_, which is independent of presented antigens and TCRs. Thus, the residence time of a T cell on an APC is determined by *k*_off_ which depends on the interaction between the presented antigen and the TCR of the T cell. Here, a smaller *k*_off_ means a greater affinity between corresponding antigen and TCR. For simplification, we assume that each APC can connect to a single T cell, which can be relaxed as discussed later.

Although there is no quantitative data on association and dissociation of T cells on APC *in vivo* at the single cell level, it is known that the affinity significantly changes depending on the combination of presented antigen and TCR. Thus, we can expect that T cells supplied to the system show a wide variety of dissociation rates for each presented antigen. Here, we assume that the distribution of dissociation rates depends only on the cell type (i.e., Tconv or Treg) and the antigen type (i.e., self or non-self), and are independent of each presented antigen. To represent a wide variety of dissociation rates, we assume that the dissociation rate *k*_off_ of supplied T cells follows a log-normal distribution, i.e., $\log_{2}\left(k_{\text{off}}^{\text{TCR,antigen}}\right)$ obeys $N\left(\mu^{\text{TCR,antigen}},\sigma^{2}\right)$ for TCR = {Tconv, Treg} and antigen = {self, non-self}, respectively, where *N*(*μ*, *σ*^2^) represents a normal distribution with mean *μ* and variance *σ*^2^. For example, $k_{\text{off}}^{\text{Tconv,self}}$ represents the dissociation rate of Tconv cells from MHC with self antigens. Here, we assume that the variance *σ*^2^ is common for all distributions of *k*_off_. Due to negative selection Tconv cells tend to have greater affinity (smaller *k*_off_) for non-self antigens than self antigens, which is represented by Δ^Tconv^ = *μ*^Tconv,self^ − *μ*^Tconv,non−self^ > 0. At the same time, we assumed that the distributions of $k_{\text{off}}^{\text{Tconv,self}}$ and $k_{\text{off}}^{\text{Tconv,non-self}}$ have a significant overlap after negative selection. To realize this assumption, we set Δ^Tconv^ = 0.75 and *σ* = 1 (The distributions are shown in Fig 1b). Furthermore, based on the experimental evidence mentioned above, we assumed that Treg cells have greater affinities for self antigens than non-self antigens, as represented by Δ^Treg^ = *μ*^Treg,non−self^ − *μ*^Treg,self^ > 0. We also assumed the affinity distributions have a significant overlap by setting Δ^Treg^ = 0.75 and *σ* = 1, as shown in Fig 1c.

Tconv and Treg cells divide only when they bind an APC. The division occurs in a stochastic manner, and the probability depends on the number of T cells on the APC. To represent the suppression of T cell proliferation by APC bound Treg cells, we assume that the division probability *D* of both Tconv and Treg cells per unit time decreases by increasing the number of Treg cells in the environment as follows:

$$\begin{array}{c} D=\frac{\alpha }{1+\beta N_{\text{Treg}}}, \end{array}$$ (1)

where *α* and *β* are constant parameters representing the basal reproduction activity and the suppression strength by Treg cells, respectively. *N*_Treg_ indicates the number of Treg cells attached to APCs in the environment. In this model, we assume that growth suppression is mediated by secretory factors [11], and neglect spatial variation of the suppression. This assumption can be relaxed, as discussed later. As a result of the cell division, two daughter T cells appear which express the same TCR as the parent. One daughter cell keeps binding to the same APC to which the parent was bound while the other daughter cell is released to the environment. As the division of a cell occurs in a stochastic manner when it attaches to an APC, a cell with a smaller dissociation rate has a larger probability of reproducing itself.

Based on the above dynamics of T cell association/dissociation and cell division, the number of T cells dynamically changes over time. Here, to calculate the dynamics of T cell association/dissociation, we adopt StochSim method [16], which is a widely used method to simulate stochastic chemical reaction dynamics. Briefly, at each time step we randomly select a pair of APC and free T cell from the environment. The selected APC is either already associated with a T cell, or has no attached T cell. When the selected APC has no attached T cell, the selected free T cell attaches to the APC with probability *k*_on_. Otherwise, the T cell already attached to the selected APC is dissociated with probability *k*_off_, which depend on the combination of the presented antigen on the APC and the TCR of the T cell. In addition to the association/dissociation dynamics of T cells, each T cell which is attached to an APC stochastically divides with the division probability *D* per time step. Furthermore, a constant number of Tconv and Treg cells with randomly chosen TCRs are supplied to the environment, while each free T cell in the environment is randomly discarded with a constant probability (see details of parameters in the caption of Fig 1). The procedure of the simulation is presented in S1 Text.

Of course, this simple model contains several simplifications and assumptions which are not validated because of the limited availability of experimental data. For example, we simply assumed that the division probability depends only on the number of Treg cells in the environment, although it is known that several other factors as secreted interleukins significantly affect the growth behavior. Here we adopt a simplified model, since at present to elucidate detailed models with realistic parameters is difficult, and the inclusion of such detailed factors can obscure an understanding of the essence of the model. Rather, in this study we start with a simplified model containing only the basic features of T cell immune response, and with this we attempt to capture the essential features of the immune system, which are independent of the details of the modeling. The generality of the results will be discussed after presenting simulation results.

## Results and Discussion

**Fig 2:**
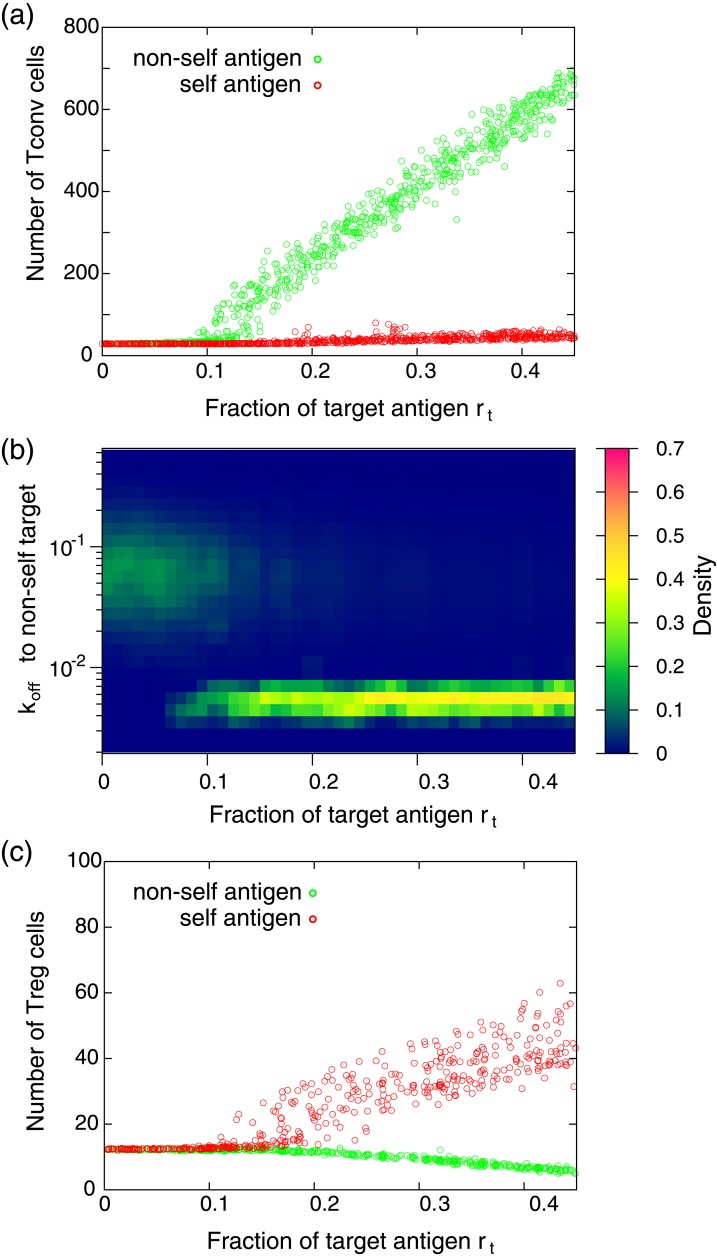
Response of T cell populations with respect to antigen presentation. (a) The average number of Tconv cells as a function of the fraction of the target antigen *r*_*t*_. Each dot represents the result obtained by presentation of different randomly chosen target antigen. The average number of Tconv cells is obtained by averaging the number over 1000 time units after the system settled down to a steady state. (b) The distribution of *k*_off_ of Tconv cells to the non-self target antigens. The distribution is obtained after the system falls into a steady state. When *r*_*t*_ is small (≲ 0.1), the distribution of *k*_off_ is almost identical to that of supplied Tconv cells to the environment (the distribution is shown in Fig 1b). In contrast, in the region of *r_t_* ≳ 0.1, the dissociation rate to the target antigen significantly decreases, indicating that T cells having high affinity to the target are selectively amplified. (c) The average number of Treg cells as a function of the fraction of the target antigen *r*_*t*_. Each dot represents the result obtained by presentation of a different target antigen. The parameters used in these calculations are *N* = 1000, *M*^self^ = *M*^non−self^ = 5000, *K*^Tconv^ = *K*^Treg^ = 5000, *k*_on_ = 0.1, *α* = 30, and *β* = 10. Tconv and Treg cells with randomly chosen TCRs are continuously supplied to the environment at a ratio of 9:1. The flow rate of T cell supply to the environment is 0.5 cell per unit time, while cells which are not attached to an APC are randomly discarded from the environment with a probability of 0.05 per unit time.

To investigate the mechanism of self/non-self discrimination, we consider the case that one randomly chosen self or non-self target antigen is presented on a certain fraction of APCs, while the other APCs present various self antigens selected randomly. Fig 2a shows the average number of Tconv cells in the system as a function of the fraction of the target antigen denoted by *r*_*t*_. The number of Tconv cells was obtained after the system falls into a steady state of the cell number. In the case of non-self antigen presentation, the number of Tconv cells sharply increases by increasing *r*_*t*_. In contrast, the number of cells is almost unchanged when self target antigen is presented. In the former case, the actively dividing Tconv cells have significantly lower *k*_off_ (higher affinity) for the presented target antigen than Tconv cells supplied from outside the system. Fig 2b shows how the distribution of *k*_off_ of Tconv for the non-self target antigens changes by increasing *r*_*t*_. As shown, *k*_off_ of Tconv cells significantly decreases when *r*_*t*_ exceed a threshold level (∼0.1). In this region, Tconv cells which have higher affinities to the target antigen are selectively amplified. The resulting population is dominated by Tconv cells which are the offspring of a few such Tconv cells. The threshold level is determined by a balance between the basal reproduction activity *α* and the suppression of reproduction by Treg cells. We confirmed that such amplification of reactive T cells to the non-self target and tolerance to the self target are independent of the choice of self and non-self target antigens.

The mechanism for the self and non-self discrimination in Fig 2 is as follows: when the fraction of the non-self target antigen increases, the affinity between Tregs and antigens on APCs decreases “on average”, due to the affinity bias Δ^Treg^. Then, the number of Tregs on APCs decreases as shown in Fig 2c, resulting in increasing the division probability *D*. As a result, a competition between T cell populations arises on the APCs, and eventually Tconv cells which have a relatively higher affinity to the target non-self antigen occupy the APCs. In contrast, when a self-antigen is presented, the number of Treg cells increases by increasing *r*_*t*_ as shown in Fig 2c. In this case, Treg cells having relatively higher affinities to the presented antigen are amplified. However, the increase of cell number is limited due to auto-suppression by the division probability. Here, the number of Tregs on APCs can be regarded as a macroscopic variable that controls the immune response, by enabling the threshold response of T cell proliferation with robust self/non-self discrimination, even though the affinity distributions to self and non-self antigens have a significant overlap.

It should be stressed that, without the regulation of cell proliferation by Treg cells, the clear self/non-self discrimination such as in Fig 2 is difficult when the negative selection is imperfect. To demonstrate this, we simulated the response to self or non-self antigen presentations when there are no Treg cells and where the affinity distribution of Tconv is identical to that in Fig 1b (Δ^Tconv^ = 0.75 and *σ* = 1). To evaluate the accuracy of self/non-self discrimination, we define the discrimination score as follows:

$$\begin{array}{c} S\equiv \int_{0}^{\frac{1}{2}}\left\{\left\langle N^{ns}\left(r_{t}\right)\right\rangle -\left\langle N^{s}\left(r_{t}\right)\right\rangle \right\}dr_{t}. \end{array}$$ (2)

**Fig 3:**
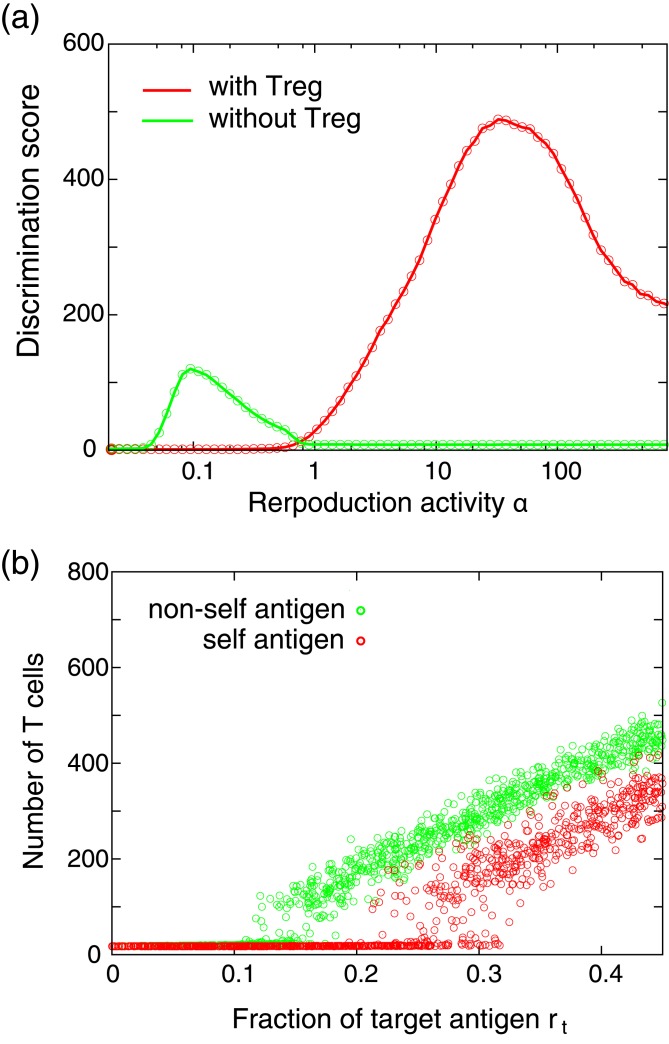
Self/non-self discrimination accuracy. (a) The discrimination score *S* as a function of basal reproduction activity *α*. (b) The average number of Tconv cells in the case without Treg regulation. The reproduction activity *α* is set to 0.1 at which the discrimination score becomes maximum. The parameters are set to those used in Fig 2, except for *α*.

Here, 〈*N*^*s*^(*r*_*t*_)〉 and 〈*N*^*ns*^(*r*_*t*_)〉 represent the average numbers of Tconv cells when a self or a non-self target antigen is presented in the ratio *r*_*t*_, respectively, where the average is taken over various target antigens. This discrimination score corresponds to the area between the curves of self and non-self antigen presentations in Fig 2a, which takes a larger value when the system can discriminate self and non-self antigens accurately. Fig 3a shows the discrimination score *S* as a function of the parameter *α* representing the basal reproduction activity. As shown in the figure, in the case without Treg cells, the discrimination score *S* has a peak around *α* ∼ 0.2. However, the maximum value of *S* is significantly smaller than in the case with Treg cells, indicating lower discrimination accuracy in the case without Treg cells. Fig 3b shows the number of Tconv cells in the case without Treg as a function of *r*_*t*_, where the parameter *α* is set to 0.1 which is close to that which results in the maximum value of *S*. As shown, the increase in Tconv cell number occurs in both cases of self and non-self antigen presentations, and thus the clear self/non-self discrimination as in Fig 2a is difficult. We have performed numerical experiments using various different parameter sets and confirmed that as long as the affinity distributions of TCRs to self and non-self antigens have a significant overlap as in Fig 1b, the maximum discrimination score is generally smaller in the case without Treg cells than in that with Treg cells, as shown in S1 Fig. The results suggested that when the affinity bias is small, clear self/non-self discrimination as in Fig 2 is possible only with the aid of Treg regulation.

**Fig 4:**
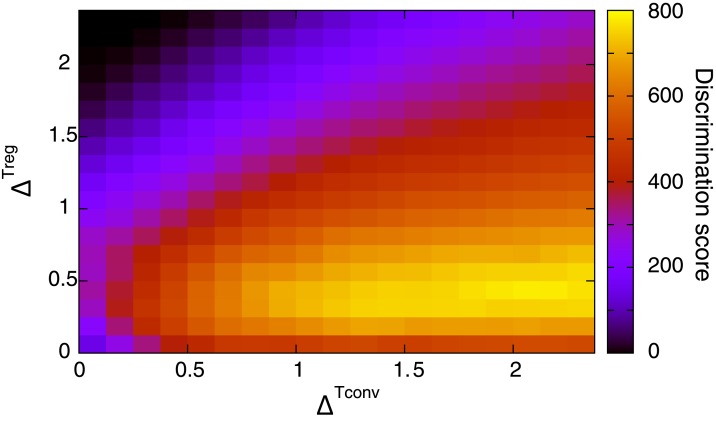
Discrimination score as a function of Δ^Tconv^ and Δ^Treg^. The color represents the maximum value of the discrimination score *S*. The maximization of *S* is taken over the basal reproduction activity *α*. *μ*^Tconv,self^ and *μ*^Treg,non−self^ are set to −3.

Fig 4 shows the maximum value of the discrimination score *S* as a function of Δ^Tconv^ and Δ^Treg^ obtained with Treg regulation. The maximization of *S* is taken over the basal reproduction activity *α*. As shown, the maximum discrimination score takes larger values in a relatively narrow range of Δ^Treg^ (e.g., 0.25 < Δ^Treg^ < 0.75). The maximum discrimination score becomes small when Δ^Treg^ is large, because in this region the active proliferation of Tconv cells is suppressed by Treg cells for both self and non-self antigen presentation. In contrast, the maximum discrimination score monotonically increases with increasing Δ^Tconv^. The dependency of discrimination performance on the affinity biases Δ^Tconv^ and Δ^Treg^ is robust on changing model rules and parameters, suggesting it is a general feature of this Treg-driven self/non-self discrimination.

**Fig 5:**
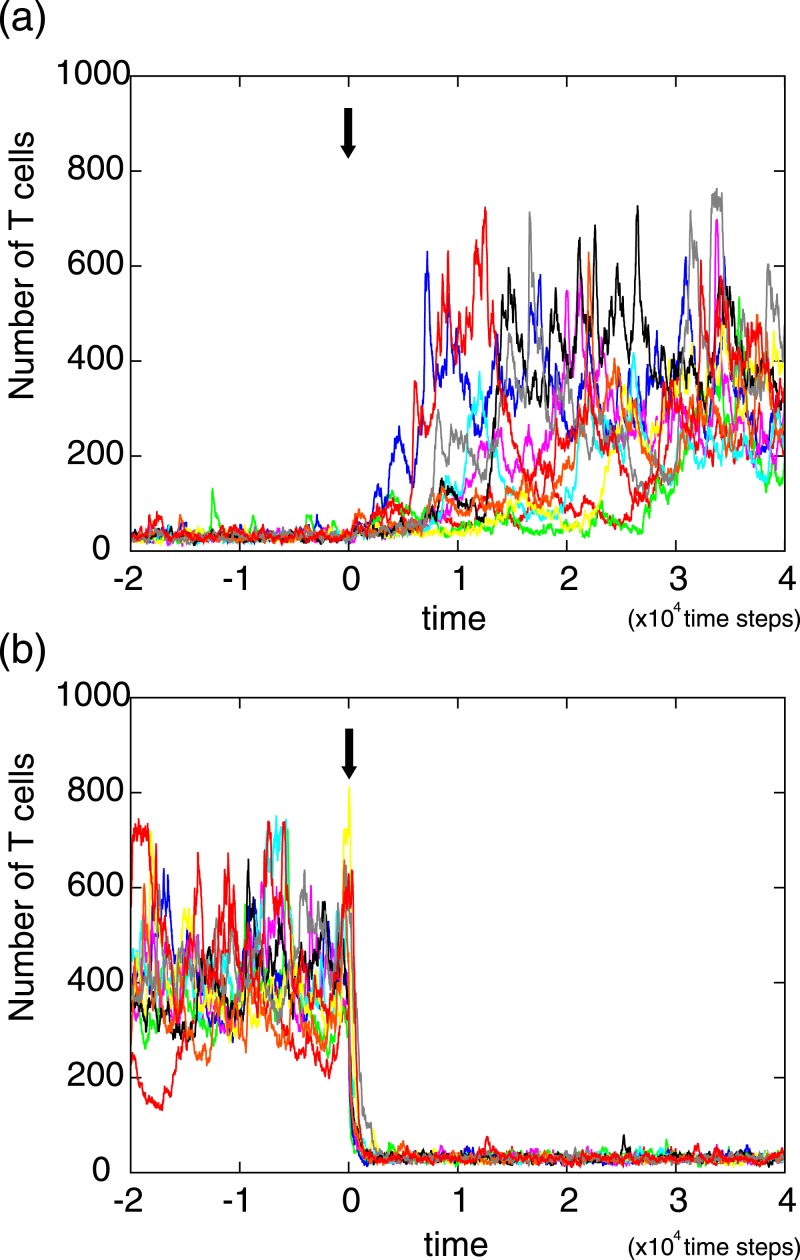
The dynamic change of T cell number in response to antigen presentation. (a) Response to a non-self antigen presentation. At time = 0 (denoted by arrow), an non-self antigen is presented in APCs with the ratio *r*_*t*_ = 0.3. In the figures, time series data obtained by 10 different initial conditions are overlaid. (b) Response to stopping a non-self antigen presentation. At time = 0, the presented non-self antigen on APCs is replaced by randomly chosen self antigens. The parameters are set to those used in Fig 2.

Fig 5 presents the dynamic change of T cell number over time in response to antigen presentation. Fig 5(a) shows the response to a non-self antigen presentation at time = 0 (denoted by arrow), in which time series data obtained by different initial conditions are overlaid. As shown, the response time to reach the new steady state fluctuates over samples. In contrast, when the non-self antigen presentation stops and all APCs start to present randomly chosen self antigens, the number of T cells quickly falls into the original steady state as shown in Fig 5(b). These results indicated that, in the former case, some time is necessary to find and to amplify Tconv cells which have high affinity to the presented non-self antigen.

To sum up, in this study we demonstrated that self/non-self discrimination is possible based on regulation by Treg cells, even when the affinity distributions of TCRs to self and non-self antigens have a significant overlap. The number of Treg cells on APCs plays the role of a macroscopic variable controlling the activation of T cells responding to presented non-self antigens. We emphasize that the results presented herein are independent of details of the modeling and valid over a broad class of models, as long as the model includes stochastic dynamics of T cell populations, the broad affinity distributions, and the suppression of T cell proliferation on APCs by Treg cells. For example, in the simulations presented in this paper, we assumed a fixed association rate constant *k*_on_ which is independent of presented antigens and TCRs. We confirmed that this assumption can be relaxed, i.e., that similar behavior of self/non-self discrimination emerges when *k*_on_ depends on the combination of antigens and TCRs, as experimental studies demonstrated [17]. Furthermore, we assumed that the affinity distribution depends only on the cell type (i.e., Tconv or Treg) and antigen type (i.e., self or non-self), and is independent of each presented antigen. Again, we confirmed that this assumption can be relaxed, i.e., even when the affinity distributions are different among presented antigens, discrimination is possible within a certain range of affinity biases, as shown in S2 Fig for example. For another example of the generality of the results, we analyzed a different model in which each APC presents multiple (e.g., ∼100) antigens and same number of Tconv or Treg cells can attach on the APC simultaneously. As shown in S3 Fig, we confirmed that robust self/non-self discrimination is also possible by this model when Treg cells suppress the proliferation of T cells which attach on the same APC, i.e., the division probability *D* of T cells on an APC is a function of the number of Treg cells on the corresponding APC. In such local suppression models, self/non-self discrimination was observed as long as the migration of T cells among APCs is active enough. Also, we relaxed the assumption that after cell division, one daughter cell keeps binding to the same APC. We performed the simulation in which both daughter cells are released into the environment after cell division event, and found that the Treg facilitated self/non-self discrimination is possible as shown in S4 Fig. However, we also found that the ranges of parameter values in which the self/non-self discrimination occurs generally shrink in this model, which might suggest the importance of sequestration of APC by T cells for the self/non-self discrimination.

There are several previous studies for theoretical analysis of the self/non-self discrimination [18]. For example, McKeithan [19] discussed that by assuming multiple reaction steps in TCR and antigen interaction, a small affinity difference between self and non-self antigens to a TCR can be amplified in a way analogous to the error reduction in kinetic proofreading [20], which can bring about an accurate self/non-self discrimination. However, this approach and many other studies generally rely on the assumption that TCRs have a greater affinity for non-self antigens than for self-antigens. This is not always the case for each combination of TCR and antigens, instead, such affinity differences should rely on the statistical difference of a huge number of combinations of TCR and antigens, as in the affinity bias shown in Fig 1b. The present study is the first to demonstrate that a small bias in the affinity distribution can maintain robust and accurate self/non-self discrimination with the suppression of T cell activation by Treg cells. Also, Freitas and colleagues discussed homeostasis of T cells by quorum-sensing mechanism [21]. Although our model neglected such complicated interactions among Tconv and Treg cells and included the simplified growth suppression by Treg cells, the inclusion of the quorum-sensing mechanism into our model might facilitate robustness of the Tconv/Treg population dynamics.

Of course, there is limited experimental support for this mechanism of discrimination. To verify the Treg-regulation based self/non-self discrimination we proposed, the most important experimental data is the affinity distribution between TCRs and self/non-self antigens. Although the affinities between TCRs and antigens have been quantified for some specific combinations, for better modeling to provide quantitative predictions, a larger number of TCR-antigen combinations should be quantified to clarify the nature of the affinity distribution and how the affinity affects the proliferation of T cells. Furthermore, the suppression of T cell proliferation by Treg cells should also be quantified to evaluate the contribution of Treg regulation to accurate self/non-self discrimination. In our model, the suppression of T cell proliferation by Treg cells was assumed to be global, i.e., the division probability of T cells depends on the total number of Treg cells attaching to APCs in the system. This is based on the assumption that secretion factors such as interleukin-10 produced by Treg cells contribute to suppression. However, in addition to such secretion factors, direct cell-cell interactions might also play a role in Treg regulation. Quantitative evaluation of the cell-cell interaction is necessary to develop more precise models describing the immune response. To obtain these quantitative data, the dynamics of T cell and APC populations should be analyzed at single-cell resolution. Recent advances in time-lapse single-cell imaging might enable us to obtain a large amount quantitative data to analyze dynamics of T cell proliferations and interactions in near future.

## Supporting Information

S1 Text Procedure of the simulation. (PDF)

S1 Fig The maximum discrimination score S with various parameter sets. The maximum values of the discrimination score S over the basal reproduction activity *α* were calculated under presence or absence of Treg cells. The sets of parameter values *k*_on_, *β*, and *μ*^Tconv,self^ were selected from uniform random distributions in [0.01, 0.3], [2, 100], and [-4, -2], respectively. *μ*^Treg,non−self^ was assumed to be identical to *μ*^Tconv,self^, while we set Δ^Tconv^ = Δ^Treg^ = 0.75. Other parameters were set to those used in Fig 2. Each point represents the maximum scores obtained by a set of randomly chosen parameters. As shown, the maximum scores generally larger in the cases with Treg cells, indicating that the accurate self/non-self discrimination can be enhanced by the suppression of T cell proliferation by Treg cells in the wide range of parameter values. The solid black line is *y* = *x* for reference.
(PDF)

S2 FigResponse of T cell populations in the case with different affinity distributions among antigens.As in Fig 2(a), the average number of Tconv cells as a function of the fraction of the target antigen *r*_*t*_ is plotted. In this simulation, the log_2_ transformed affinities between TCRs on Tconv or Treg cells and *i*-th antigen obeys $N(\mu_{i}^{\text{Tconv}},\sigma^{2})$ and $N(\mu_{i}^{\text{Treg}},\sigma^{2})$, respectively. Here, $\mu_{i}^{\text{Tconv}}$ and $\mu_{i}^{\text{Treg}}$ depend the antigens, and we assumed that they obey $N(\bar{\mu^{\text{Tconv,self}}},0.1^{2})$ and $N(\bar{\mu^{\text{Treg,self}}},0.1^{2})$ for self antigens, and $N(\bar{\mu^{\text{Tconv,non-self}}},0.1^{2})$ and $N(\bar{\mu^{\text{Treg,non-self}}},0.1^{2})$ for non-self antigens, respectively. We used $\bar{\mu^{\text{Tconv,self}}}=\bar{\mu^{\text{Treg,non-self}}}=-3$ and $\bar{\mu^{\text{Tconv,non-self}}}=\bar{\mu^{\text{Treg,self}}}=-3.75$, respectively. As shown, even when the affinity distributions are different among presented antigens, discrimination is possible.(PDF)

S3 FigResponse of T cell populations in the case when multiple antigens are presented on each APC.In this simulation, 50 antigens are presented on each APC, and the number of APCs in the environment is fixed on 4. In the figure, the average number of Tconv cells attached to one APC is plotted as a function of the fraction of the target antigen *r*_*t*_. (a) and (b) show the average number of Tconv cells in the case with and without Treg regulation, respectively. The T cell division probability on *j*-th APC follows $D_{j}=\alpha /\left(1+\beta N_{\text{Treg}}^{j}\right)$, where $N_{\text{Treg}}^{j}$ represents the number of Treg cells attached to *j*-th APC. The reproduction activity *α* determined to those which maximize the discrimination score. Other parameter are set to those used in Fig 2.(PDF)

S4 Fig Response of T cell populations in the case when both daughter cells are released after cell division. In this simulation, the flow rate of T cell supply to the environment is set to 0.1 cell per unit time, while cells while cells which are not attached to an APC are randomly discarded from the environment with a probability of 0.01 per unit time. The other parameter values are set to those used in Fig 2.
(PDF)

## References

[1] Guermonprez P, Valladeau J, Zitvogel L, Thery C, Amigorena S. Antigen presentation and T cell stimulation by dendritic cells. Ann Rev Immunol. 2002; 20(1):621–667. 10.1146/annurev.immunol.20.100301.064828 11861614

[2] Nikolich-Zugich J, Slifka MK, Messaoudi I. The many important facets of T cell repertoire diversity. Nat Rev Immunol. 2004; 4(2): 123–132. 10.1038/nri1292 15040585

[3] Kieper WC, Burghardt JT, Surh CD. A role for TCR affinity in regulating naive T cell homeostasis. Jour Immunol. 2004; 172(1): 40–44. 10.4049/jimmunol.172.1.40 14688307

[4] Surh CD, Sprent J. T cell apoptosis detected in situ during positive and negative selection in the thymus. Nature. 1994; 372(6501): 100–103. 10.1038/372100a0 7969401

[5] Walker LS, Abbas AK. The enemy within: keeping self-reactive T cells at bay in the periphery. Nat Rev Immunol. 2002; 2(1): 11–19. 10.1038/nri701 11908514

[6] Sakaguchi S, Yamaguchi T, Nomura T, Ono M. Regulatory T cells and immune tolerance. Cell. 2008; 133(5): 775–787. 10.1016/j.cell.2008.05.009 18510923

[7] Sakaguchi S, Sakaguchi N, Asano M, Itoh M, Toda M. Immunologic self-tolerance maintained by activated T cells expressing IL-2 receptor alpha-chains (cd25). breakdown of a single mechanism of self-tolerance causes various autoimmune diseases. Jour Immunol. 1995; 155(3): 1151–64. 7636184

[8] Yamaguchi T, Hirota K, Nagahama K, Ohkawa K, Takahashi T, Nomura T, Sakaguchi S. Control of immune responses by antigen-specific regulatory T cells expressing the folate receptor. Immunity. 2007; 27(1):145–59. 10.1016/j.immuni.2007.04.017 17613255

[9] Josefowicz SJ, Lu LF, Rudensky AY. Regulatory T cells: mechanisms of differentiation and function. Annu Rev Immunol. 2012; 30:531–64. 10.1146/annurev.immunol.25.022106.141623 22224781 PMC6066374

[10] Hsieh CS, Lee HM, Lio CW. Selection of regulatory T cells in the thymus. Nat Rev Immunol. 2012; 12(3):157–167. 22322317 10.1038/nri3155

[11] Shevach EM. Mechanisms of Foxp3+ T regulatory cell-mediated suppression. Immunity. 2009; 30(5):636–45. 10.1016/j.immuni.2009.04.010 19464986

[12] Yates A, Bergmann C, Van Hemmen JL, Stark J, Callard R. Cytokine-modulated regulation of helper T cell populations. Jour Theor Biol. 2000; 206(4): 539–560. 10.1006/jtbi.2000.2147 11013114

[13] Carneiro J, Leon K, Caramalho I, van den Dool C, Gardner R, Oliveira V, et al When three is not a crowd: a crossregulation model of the dynamics and repertoire selection of regulatory CD4+ T cells. Immunol Rev. 2007; 216(1):48–68. 10.1111/j.1600-065X.2007.00487.x 17367334

[14] Hong T, Xing J, Li L, Tyson JJ. A mathematical model for the reciprocal differentiation of T helper 17 cells and induced regulatory T cells. PLoS Comp Biol. 2011; 7(7): e1002122 10.1371/journal.pcbi.1002122 PMC3145653 21829337

[15] Khailaie S, Bahrami F, Janahmadi M, Milanez-Almeida P, Huehn J, Meyer-Hermann M. A mathematical model of immune activation with a unified self-nonself concept. Front Immunol. 2013; 4: 474 10.3389/fimmu.2013.00474 24409179 PMC3872974

[16] Morton-Firth CJ, Bray D. Predicting Temporal Fluctuations in an Intracellular Signalling Pathway. Jour Theor Biol. 1998; 192: 117–128. 10.1006/jtbi.1997.0651 9628844

[17] Huang J, Zarnitsyna VI, Liu B, Edwards LJ, Jiang N, Evavold BD, Zhu C. The kinetics of two-dimensional TCR and pMHC interactions determine T-cell responsiveness. Nature. 2010; 464: 932–936. 10.1038/nature08944 20357766 PMC2925443

[18] Carneiro J, Paixao T, Milutinovicb D, Sousaa J, Leona K, Gardnera R, Faroa J. Immunological self-tolerance: Lessons from mathematical modeling. Jour Comp Appl Math. 2005; 184: 77–100. 10.1016/j.cam.2004.10.025

[19] Mckeithan TW. Kinetic proofreading in T cell receptor signal transduction. Proc Nat Acad Sci. 1995; 92(11): 5042–5046. 10.1073/pnas.92.11.5042 7761445 PMC41844

[20] Hopfield JJ. Kinetic proofreading: a new mechanism for reducing errors in biosynthetic processes requiring high specificity. Proc Nat Acad Sci. 1974; 71(10): 4135–4139, 1974 10.1073/pnas.71.10.4135 4530290 PMC434344

[21] Almeida AR, Amado IF, Reynolds J, Berges J, Lythe G, Molina-Par?s C, Freitas AA. Quorum-sensing in *CD*4^+^ T cell homeostasis: a hypothesis and a model. Front Immun. 2012; 3: 125 10.3389/fimmu.2012.00125 PMC3360200 22654881

