# Supplementary material for: Robust and Accurate Discrimination of Self/Non-Self Antigen Presentations by Regulatory T Cell Suppression
Source: PLoS One. 2016 Sep 26;11(9):e0163134. doi: 10.1371/journal.pone.0163134 (PMC5036821; doi:10.1371/journal.pone.0163134)
Supplement: S1 Text — (PDF) [file pone.0163134.s001.pdf]

S1 Text: Procedure of the simulation

In this supporting text, we describe the procedure of the simulation shown in Fig. 2.

Step 1: Setting the parameter values.
The parameter values are set as follows:
the number of APCs in the environment $N = 1000$,
the number of self antigen species $M^{\mathrm{self}} = 5000$,
the number of non-self antigen species $M^{\mathrm{non-self}} = 5000$,
the number of TCR species on Tconv $K^{\mathrm{Tconv}} = 5000$,
the number of TCR species on Treg $K^{\mathrm{Treg}} = 5000$,
the association rate between APC and Tcell $k_{\mathrm{on}} = 0.1$,
the basal reproduction activity $\alpha = 30$, and
the suppression strength by Treg cells $\beta = 10$.

Step 2: Setting the dissociation rate k_{off} between antigens and APCs.
Let us consider $K^{\mathrm{Tconv}} \times M^{\mathrm{self}}$ matrix representing $k_{\mathrm{off}}^{\mathrm{Tconv,self}}$, in which the value at i-th column and j-th row represents $k_{\mathrm{off}}^{\mathrm{Tconv,self}}$ between i-th TCR on Tconv and j-th self antigen in the repertoires. These values are determined randomly. Here, we assumed that they follows a log-normal distribution, i.e., $\log_2(k_{\mathrm{off}}^{\mathrm{Tconv,self}})$ obeys $N(\mu^{\mathrm{Tconv,self}}, \sigma^2)$, where $N(\mu^{\mathrm{Tconv,self}}, \sigma^2)$ represents a normal distribution with mean $\mu^{\mathrm{Tconv,self}}$ and variance σ^2. Similarly, $k_{\mathrm{off}}^{\mathrm{Tconv,non-self}}$, $k_{\mathrm{off}}^{\mathrm{Treg,self}}$, and $k_{\mathrm{off}}^{\mathrm{Treg,non-self}}$ are determined with the parameters $\mu^{\mathrm{Tconv,non-self}}$, $\mu^{\mathrm{Treg,self}}$, $\mu^{\mathrm{Treg,non-self}}$, respectively. In the simulation shown in Fig.2, we use $\mu^{\mathrm{Tconv,self}} = \mu^{\mathrm{Tconv,non-self}} = -3$ and $\mu^{\mathrm{Tconv,non-self}} = \mu^{\mathrm{Treg,self}} = -3.75$, respectively. The standard deviation σ is fixed on 1 for all distributions (the distributions of k_{off} are shown in Fig.1(b) and (c)).

Step3: Setting the initial conditions.
Prepare N APCs in the environment, each of which presents one antigen. Then, choose one self or non-self target antigen from repertoires of antigens at random. A certain fraction of APCs present the target antigen (r_t is the ratio of APCs presenting the target antigen to the total APCs), while the other APCs present randomly chosen self antigens from M^{self} repertory. The number of APCs and antigens presented by them are fixed throughout the simulation. The initial number of free T cells in the environment is set to N. The number

ratio of Tconv to Treg cells in the initial population is set to 9 : 1. The TCRs on Tconv and Treg cells are determined randomly from K^{Tconv} and K^{Treg} repertoires, respectively. Set the time count $t = 0$.

Step 4: Checking association/dissociation between APC and T cell.
Randomly select a pair of APC and free T cell (Tconv or Treg) from the environment. If the selected APC has no attached T cell, the selected free T cell attaches to the APC with probability k_{on}. If the APC already has attached T cell on it, the attached T cell is dissociated with probability k_{off}, which depends on the pair of antigen on the APC and TCR on the T cell (k_{off} values for all possible combinations of TCR and antigen are already determined in Step 2). The dissociated T cell is added to the population of free T cells in the environment.

Step 5: Checking cell division.
Calculate the total number of Treg cells, N_{Treg}, which are already attached to APCs in the environment. Then, randomly select an APC. If a T cell is already attached to the selected APC, this T cell divides with the probability $D = \frac{\alpha}{1+\beta N_{\mathrm{Treg}}}$. The division probabilities of Tconv and Treg cells are assumed to be identical. The two daughter T cells have the identical TCR to the parent T cell. One daughter T cell keeps attachment to the APC, while the other T cell is released to the environment and becomes a free T cell.

Step 6: Checking supply and discard of T cells to/from the environment.
In the simulation shown in Fig. 2, 0.5 T cell is supplied to the environment per unit time. That is, for each two time steps, one Tconv or Treg cell is added to the population of free T cells. The number ratio of Tconv to Treg cells in these supplied T cells is set to 9 : 1. The TCRs on the supplied Tconv or Treg cells are determined randomly from K^{Tconv} or K^{Treg} repertoires. At the same time, a certain fraction of free T cells are discarded from the environment. In the simulation shown in Fig. 2, each free T cell is discarded with the probability of 0.05 for each time step.

Step 7: Set $t = t + 1$ and go to step 4.
The data shown in Fig. 2 was obtained after the number of T cells falls into a steady state (e.g., $t > 10^5$).

Note:

The above Step 1 to Step 7 presents the procedure for the simulation shown in Fig.2. The assumptions used in the procedure can be relaxed as discussed in the 6th paragraph in Results and Discussion section. For such simulations the above procedures should be modified accordingly.
