# Supplementary figures and images for: Robust and Accurate Discrimination of Self/Non-Self Antigen Presentations by Regulatory T Cell Suppression
Source: PLoS One. 2016 Sep 26;11(9):e0163134. doi: 10.1371/journal.pone.0163134 (PMC5036821; doi:10.1371/journal.pone.0163134)

Supporting Figure S1

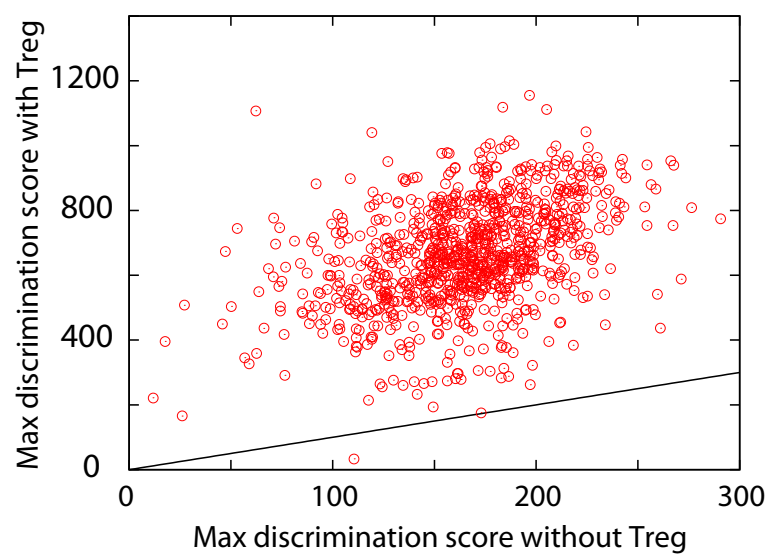

Supplement: S1 Fig — The maximum values of the discrimination score S over the basal reproduction activity α were calculated under presence or absence of Treg cells. The sets of parameter values kon, β, and μTconv,self were selected from uniform random distributions in [0.01, 0.3], [2, 100], and [-4, -2], respectively. μTreg,non−self was assumed to be identical to μTconv,self, while we set ΔTconv = ΔTreg = 0.75. Other parameters were set to those used in Fig 2. Each point represents the maximum scores obtained by a set of randomly chosen parameters. As shown, the maximum scores generally larger in the cases with Treg cells, indicating that the accurate self/non-self discrimination can be enhanced by the suppression of T cell proliferation by Treg cells in the wide range of parameter values. The solid black line is y = x for reference. (PDF) [file pone.0163134.s002.pdf]

Supporting Figure S2

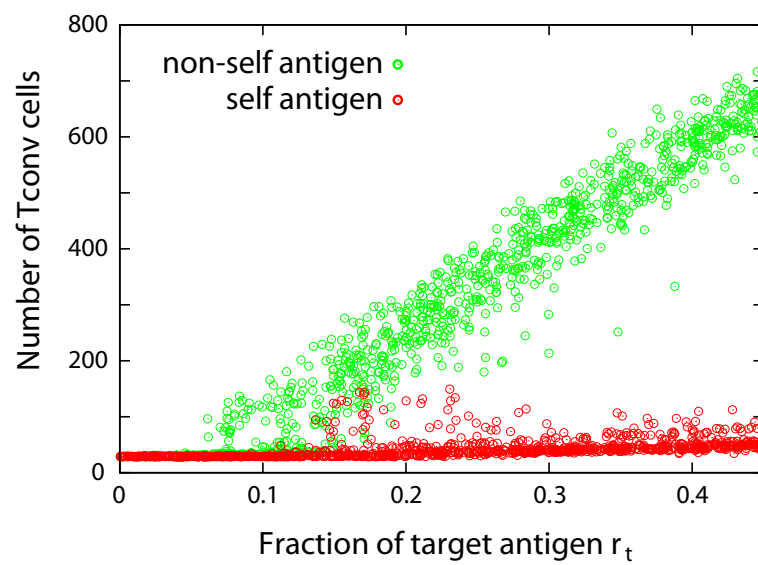

Supplement: S2 Fig — As in Fig 2(a), the average number of Tconv cells as a function of the fraction of the target antigen rt is plotted. In this simulation, the log2 transformed affinities between TCRs on Tconv or Treg cells and i-th antigen obeys N(μiTconv,σ2) and N(μiTreg,σ2), respectively. Here, μiTconv and μiTreg depend the antigens, and we assumed that they obey N(μTconv,self¯,0.12) and N(μTreg,self¯,0.12) for self antigens, and N(μTconv,non-self¯,0.12) and N(μTreg,non-self¯,0.12) for non-self antigens, respectively. We used μTconv,self¯=μTreg,non-self¯=-3 and μTconv,non-self¯=μTreg,self¯=-3.75, respectively. As shown, even when the affinity distributions are different among presented antigens, discrimination is possible. (PDF) [file pone.0163134.s003.pdf]

Supporting Figure S3

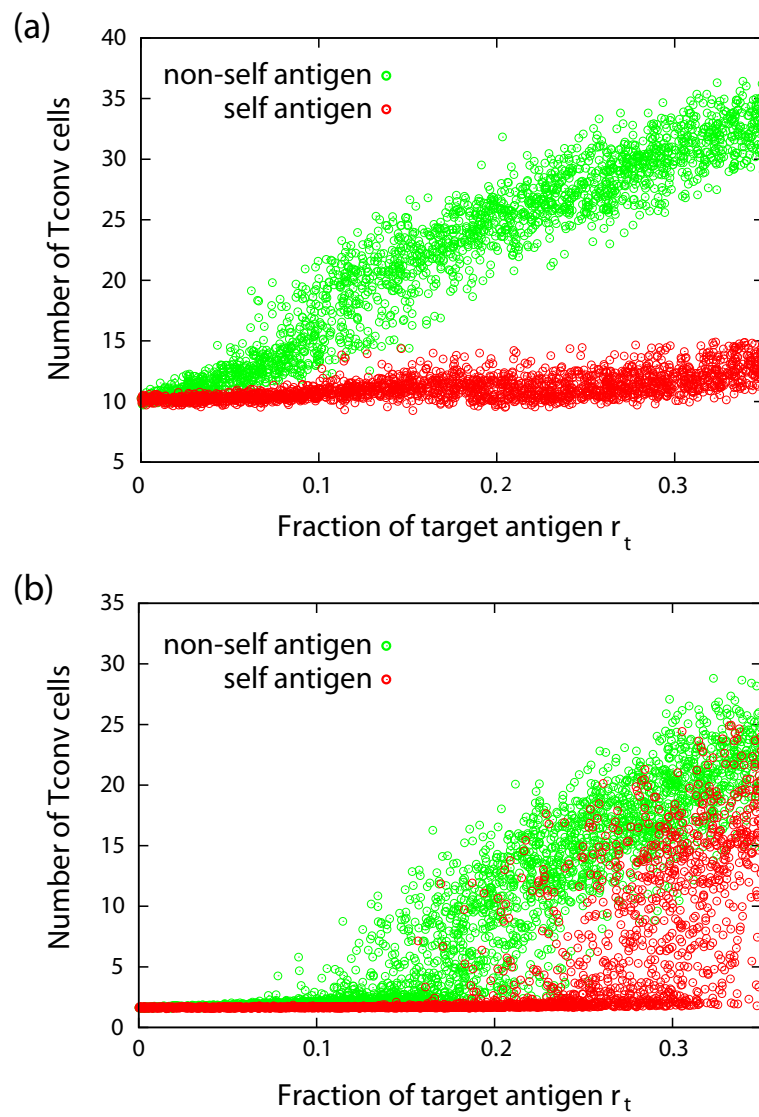

Supplement: S3 Fig — In this simulation, 50 antigens are presented on each APC, and the number of APCs in the environment is fixed on 4. In the figure, the average number of Tconv cells attached to one APC is plotted as a function of the fraction of the target antigen rt. (a) and (b) show the average number of Tconv cells in the case with and without Treg regulation, respectively. The T cell division probability on j-th APC follows Dj=α/(1+βNTregj), where NTregj represents the number of Treg cells attached to j-th APC. The reproduction activity α determined to those which maximize the discrimination score. Other parameter are set to those used in Fig 2. (PDF) [file pone.0163134.s004.pdf]

Supporting Figure S4

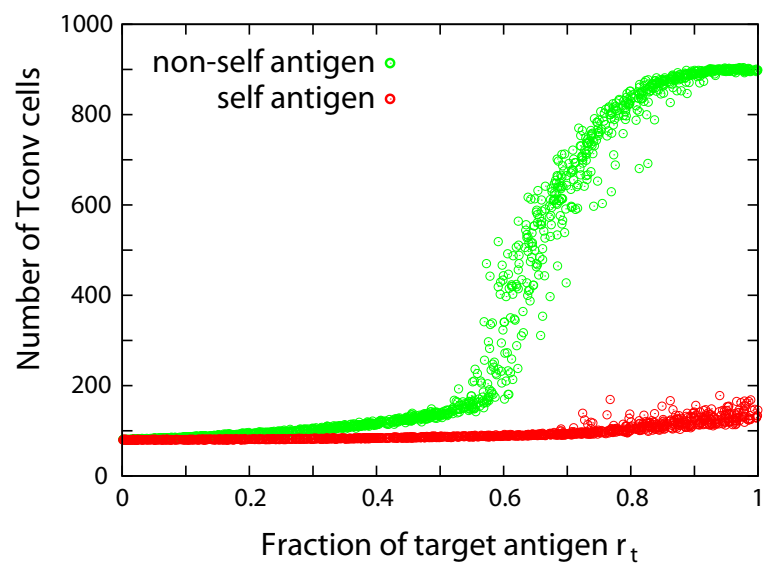

Supplement: S4 Fig — In this simulation, the flow rate of T cell supply to the environment is set to 0.1 cell per unit time, while cells while cells which are not attached to an APC are randomly discarded from the environment with a probability of 0.01 per unit time. The other parameter values are set to those used in Fig 2. (PDF) [file pone.0163134.s005.pdf]
